# A Diagnostic Triad in the Vesicular Stage of Incontinentia Pigmenti

**DOI:** 10.34172/aim.34777

**Published:** 2025-09-01

**Authors:** Thien Nguyen, Tuan Anh Vu

**Affiliations:** ^1^Emergency Resuscitation Department, Quy Hoa National Dermatology Hospital, South Quy Nhon, Gia Lai, Vietnam; ^2^Board of Directors, Quy Hoa National Dermatology Hospital, South Quy Nhon, Gia Lai, Vietnam

**Figure 1:**
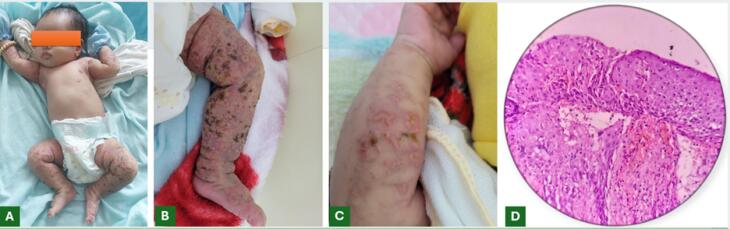
**(A, B, C)** The clinical presentation revealed vesicles and tense bullae on an erythematous base, distributed along an apparent Blaschko-linear pattern predominantly involving the patient’s extremities; **(D)** The histopathology slide demonstrated prominent eosinophilic spongiosis with intraepidermal vesicles containing eosinophils.

 A 2-month-old female infant presented with cutaneous symptoms since birth, characterized by vesicles and tense bullae on an erythematous base. The lesions seem to exhibit a Blaschko-linear distribution predominantly involving the extremities (Figure 1A, B and C). The infant was hemodynamically stable, alert, feeding well, afebrile, and without irritability. Clinical examination revealed no extracutaneous abnormalities. Family history revealed similar cutaneous symptoms during infancy in the patient’s mother, maternal aunt, and maternal grandmother. Complete blood count demonstrated leukocytosis with eosinophilia dominance. Histopathology of lesions revealed prominent eosinophilic spongiosis (Figure 1D).

 The vesicular stage is the first stage of incontinentia pigmenti (IP), a disease with four distinct stages, each corresponding to the individual’s growth. The estimated incidence of IP is approximately 0.7 cases per 100 000 births.^1^ As an X-linked dominant genetic disorder, IP manifests predominantly in females because affected males typically cannot survive until birth. The characteristic clinical symptoms of vesicular stage IP include vesicles and tense bullae on an erythematous base, distributed along Blaschko lines.^2^ Diagnosing IP is generally straightforward but may pose challenges for less experienced physicians. When clinical assessment is inconclusive, supportive diagnostic tools include complete blood count demonstrating eosinophil-predominant leukocytosis and histopathological evidence of eosinophilic spongiosis. Family history evaluation is also crucial, particularly investigating dermatological conditions in female relatives on the maternal side of the patient’s family and documentation of miscarriages or absence of male offspring in the maternal lineage. In most cases, the diagnosis of vesicular stage IP still relies primarily on cutaneous manifestations.^3^ This principle is reflected in the 2014 diagnostic criteria,^4^ which require at least one major criterion, characteristic stage 1 skin findings, and at least one minor criterion, frequently histopathological evidence of eosinophilic spongiosis, particularly when extracutaneous involvement is relatively subtle or goes unnoticed in otherwise healthy infants like our case.

 In summary, the triad of characteristic skin lesions, peripheral eosinophilia, and histopathological eosinophilic spongiosis can be considered the cornerstone for diagnosing the vesicular stage of IP.
